# Effects of Sex and Race on Epidemiology and Comorbidities of Patients with Irritable Bowel Syndrome: A Rome III Era Retrospective Study

**DOI:** 10.3390/diseases13050161

**Published:** 2025-05-21

**Authors:** Jacqueline Liu, Kathleen Cheng, Yu Lu, Howard Cabral, Horst Christian Weber

**Affiliations:** 1 Department of Medicine, Boston Medical Center, Boston, MA 02118, USA; 2 Department of Biostatistics, Boston University School of Public Health, Boston, MA 02118, USA; luyu773@bu.edu (Y.L.); hjcab@bu.edu (H.C.); 3 Section of Gastroenterology, Boston University Chobanian & Avedisian School of Medicine, Boston, MA 02118, USA; 4 VA Boston Healthcare System, Medicine Service, Section of Gastroenterology, Boston, MA 02130, USA

**Keywords:** irritable bowel syndrome, sex, comorbidities, depression, anxiety, racial differences, eating disorders

## Abstract

Background: Irritable bowel syndrome (IBS) is a prevalent disorder of gut–brain interaction (DGBI) with a negative impact on quality of life and healthcare expenditure. This study aimed to investigate sex-based differences in a large cohort of IBS patients from a multiracial safety-net hospital. Methods: An electronic query was performed using the International Classification of Diseases, 9th Revision (ICD-9) coding to identify 740 outpatients with IBS between 1 January 2005 and 30 September 2007. Demographic data and ICD-9 coded comorbidities were extracted from electronic records. Data analysis used descriptive statistics and multiple logistic regression analyses. Results: Comorbid anxiety and depression were significantly more prevalent in female patients (A:24%, *p* = 0.03; D:29%, *p* = 0.008) compared with male patients. White female IBS patients had a higher risk for anxiety but not depression compared with non-White patients (*p* = 0.02). Female sex (*p* = 0.02), obesity (*p* = 0.007), and age above fifty (*p* = 0.02) but not race/ethnicity were significant risk factors for depression. IBS with constipation was more prevalent in female patients (*p* = 0.005) and in Hispanic compared with non-Hispanic patients (*p* = 0.03). Conclusions: Significant sex-based and racial/ethnic differences were identified related to body mass index (BMI), age, and IBS subtypes in this study. Comorbid mood disorders occurred significantly more frequently in female patients, and risk factors for comorbid depression included female sex, older age, and obesity but not race/ethnicity.

## 1. Introduction

Irritable bowel syndrome (IBS) is a highly prevalent disorder of gut–brain interaction (DGBI), previously termed functional gastrointestinal disorders (FGIDs), which manifests through recurring episodes of abdominal pain associated with altered bowel habits in the absence of organic or structural abnormalities [1,2,3]. IBS is clinically diagnosed using the Rome criteria, a symptom-based criteria initially established in the late 1980s by consensus among experts in the field of functional gastrointestinal disorders [4]. In the most current iteration, the Rome IV criteria, IBS is categorized into four subtypes based upon patterns of bowel habits and include the following: IBS with diarrhea (IBS-D), IBS with constipation (IBS-C), IBS with mixed bowel habits (IBS-M), and un-subtyped IBS (IBS-U) [3,4].

Epidemiological studies have indicated that IBS symptoms affect approximately 4.7% to 5.3% of the global population and an estimated 6.1% of the population of the United States (U.S.) based on the Rome IV criteria [5,6,7,8]. This chronic and often debilitating disorder has a considerable impact on healthcare expenditure and patient quality of life [9]. IBS imposes a significant burden to the U.S. healthcare system, with a recent study estimating the total annual costs for patients with IBS at a median of USD 13,288 compared with USD 5999 for non-IBS controls [10]. Of note, healthcare spending varied with sex and IBS-subtype; female sex and IBS-C subtype were factors associated with significantly higher all-cause and IBS-specific costs [10,11]. In addition to increased healthcare utilization, IBS also indirectly contributes to economic burden through loss of work productivity [12].

The overall detrimental effect of IBS on patients’ quality of life has been well-documented. In a systematic review assessing health-related quality of life (HRQoL) using the Short Form 36 Health Survey (SF-36), patients with IBS reported significantly lower scores compared with controls across all domains including physical functioning, bodily pain, social functioning, and mental health [13]. Other studies highlighted that scores on several SF-36 scales were significantly lower in IBS patients compared with patients with chronic diseases such as asthma, gastroesophageal reflux disease (GERD), diabetes mellitus, end-stage renal disease (ESRD), and migraines [14,15].

IBS has been associated with various gastrointestinal and extraintestinal comorbidities including other gastrointestinal disorders, somatic pain syndromes, and psychiatric conditions. In a systematic review, Whitehead et al. identified fibromyalgia, chronic fatigue syndrome, chronic pelvic pain, and temporomandibular joint disorder as common somatic conditions in patients with IBS [16]. Another study highlighted an increased prevalence of chronic pain and diabetes mellitus in IBS patients compared with the control [17]. Similarly, in our prior work, we demonstrated that GERD, chronic obstructive pulmonary disease (COPD)/asthma, obesity, depression, and anxiety were conditions that frequently co-occurred in patients with IBS [18]. In recent years, there has been a growing body of literature supporting the association between IBS and psychiatric comorbidities, particularly anxiety and depression [19,20,21,22,23]. Studies have indicated that stress, anxiety, and depression may impact the severity of IBS symptoms through alterations of the neuro-endocrine-immune pathways, thereby influencing intestinal motility, permeability, and visceral sensitivity [24,25,26]. There are also some data to suggest that the IBS-C subtype may be associated with increased anxio-depressive symptomatology compared with other subtypes [27], although other studies found no significant difference between subtypes [28,29].

As a consensus across various epidemiological studies, IBS is recognized as a female-predominant disease [6,30]. A meta-analysis of thirty studies revealed that the pooled prevalence of IBS was 12% for female patients (95% CI, 9.3% to 15%) and 8.6% for male patients (95% CI, 6.3% to 11.2%) [30]. The underlying mechanism responsible for this sex-based discrepancy in IBS remains largely unknown, but prior studies have examined sex differences in the hypothalamic–pituitary–adrenal (HPA) axis activation, various cytokine profiles, and gut microbiota composition in IBS patients [26]. One study noted the presence of increased circulating pro-inflammatory cytokines (IL-17 and TNFα) and decreased anti-inflammatory cytokines (IL-10) in female patients, which may contribute to mucosal inflammation and increased IBS severity in female patients [31]. Another study suggested that variations in gut microbiota, such as increased levels of the Bacteroides-Prevotella group in male patients, may lead to differences in low-grade inflammation, immune dysfunction, and IBS susceptibility [26]. However, there is a significant paucity of data regarding differences in the sex-based associations of common IBS comorbidities in male and female patients from different racial/ethnic groups. Therefore, our study aimed to address this knowledge gap by identifying potential sex-related differences in common gastrointestinal, extraintestinal, and psychiatric conditions at a large multiracial/ethnic academic safety-net hospital.

## 2. Materials and Methods

### 2.1. Study Design

This cross-sectional retrospective study was conducted at the Boston Medical Center (BMC), an urban academic medical center that is recognized as the largest tertiary safety-net hospital in New England. The study was approved by the BMC Institutional Review Board (IRB) and was in accordance with the ethical standards of the institutional and national research committee as well as the 1995 Helsinki Declaration and its later amendments or comparable ethical standards. Due to the nature of this retrospective study and the preserved anonymity of patients, a waiver of informed consent was obtained from and approved by the BMC IRB.

Between 1 January 2005 and 30 September 2007, a total of 740 patients aged eighteen years and older were identified by performing an electronic query of the International Classification of Diseases, 9th Revision (ICD-9) code for the diagnosis of irritable bowel syndrome (564.1) as previously reported [18,32]. In the United States, the Department of Health and Human Services (HHS) mandated the official transition from ICD-9 to ICD-10 on 1 October 2015 [33], although this transition may have occurred earlier at various institutions. During the time of enrollment for this study, the ICD-9 code set was exclusively used for the BMC electronic medical records system and the ICD-10 code set was not yet active. Patients were retrospectively enrolled in reverse chronological order, beginning on 30 September 2007 and ending with 1 January 2005, at which time there was a sufficient number of patients enrolled for data analysis. Patient enrollment was based exclusively on ICD-9 coding and included all eligible patients. IBS comorbidities were also identified using ICD-9 coding. An electronic chart review was performed to categorize patients under the four-subtype classification system (IBS-C, IBS-D, IBSM, and IBS-U) based on documented symptoms and the Rome III criteria, which was the most updated IBS clinical criteria during the study period. The inter-rater agreement between the IBS subtype classifications by the research gold standard and investigators reviewing all charts was assessed (*n* = 20 charts) using Cohen’s kappa coefficient, (κ = 0.77), indicating substantial agreement.

### 2.2. Statistical Methods

In order to examine the univariate distributions of the data, we generated descriptive statistics using counts and percentages for the categorical variables (e.g., gender, race, insurance type) and medians, interquartile ranges, and standard deviations for the continuous variables (e.g., age, body mass index (BMI)). Next, we performed bivariate analyses using chi-square tests and Fisher’s exact tests for the cross-tabulations of categorical variables including comparisons of sex by BMI category and insurance type. For the continuous variables, independent *t*-tests and Wilcoxon rank-sum tests were used to compare the medians and means by sex groups, focusing on variables such as age and BMI. Additionally, one-way analyses of variance (ANOVA) were used for continuous outcomes with more than two groups (e.g., age by BMI category), with post hoc comparisons following significant global tests using Tukey’s studentized range procedure.

To further explore associations, logistic regression analyses were conducted to evaluate the relationship between demographic variables (e.g., gender, race, BMI) and outcomes such as anxiety, depression, and constipation, with interaction terms included to explore race and gender effects. Multivariate models were adjusted for potential confounders, including employment status and insurance type, to assess associations between race and IBS subtypes, with stratification by Hispanic versus non-Hispanic ethnicity. All analyses were performed using SAS software, version 9.4 (SAS Institute Inc., Cary, NC, USA), and *p*-values less than 0.05 were considered statistically significant. In our logistic regression modeling, all independent variables were entered into the model simultaneously rather than stepwise or individually. Our goal was to adjust for known covariates and assess their associations in a fully adjusted model. 

## 3. Results

### 3.1. IBS Study Population and Demographic Characteristics

Of the 740 patients with IBS enrolled in the study, there was a 3:1 ratio of female to male patients, with 555 female and 185 male patients. The entire cohort of 740 patients was grouped by race or ethnicity according to then existing guidelines [34], resulting in 492 White patients (70%), 99 Black patients (14%), 65 Hispanic patients (9%), and 44 Asian patients (6%). There was one Native American patient (*n* = 1); this patient was not included in analyses where race/ethnicity were considered because of the small number. Thirty-nine patients did not report their race/ethnicity. Therefore, analyses based on race/ethnicity were conducted in a cohort of 700 patients. The demographic characteristics of all patients are shown in Table 1. There was a significant difference in the current age of IBS patients, with female patients more likely to present below the age of fifty compared with male patients (*p* = 0.03). Female IBS patients on average had lower BMI, with a substantial majority of male patients categorized as overweight/ obese, whereas only half of the female patients were found to be overweight/ obese (*p* = 0.01). Compared with the male patients, female IBS patients were more likely to be current smokers (*p* = 0.03). Although there were several data points not reported for the highest level of education, male IBS patients were more likely than female patients to hold graduate/professional degrees (*p* = 0.005). Similarly, a large percentage of patients omitted employment information, and the unspecified employment category also included students. No significant sex-related differences were identified for race/ethnicity, marital status, children status, or insurance type (Table 1). The racial/ethnic composition of the IBS cohort differed significantly from that of the unique annual general outpatient population at Boston Medical Center during the years of the study at hand, as we previously reported [32].

### 3.2. Psychiatric Comorbidities in IBS Patients

**Figure 1:**
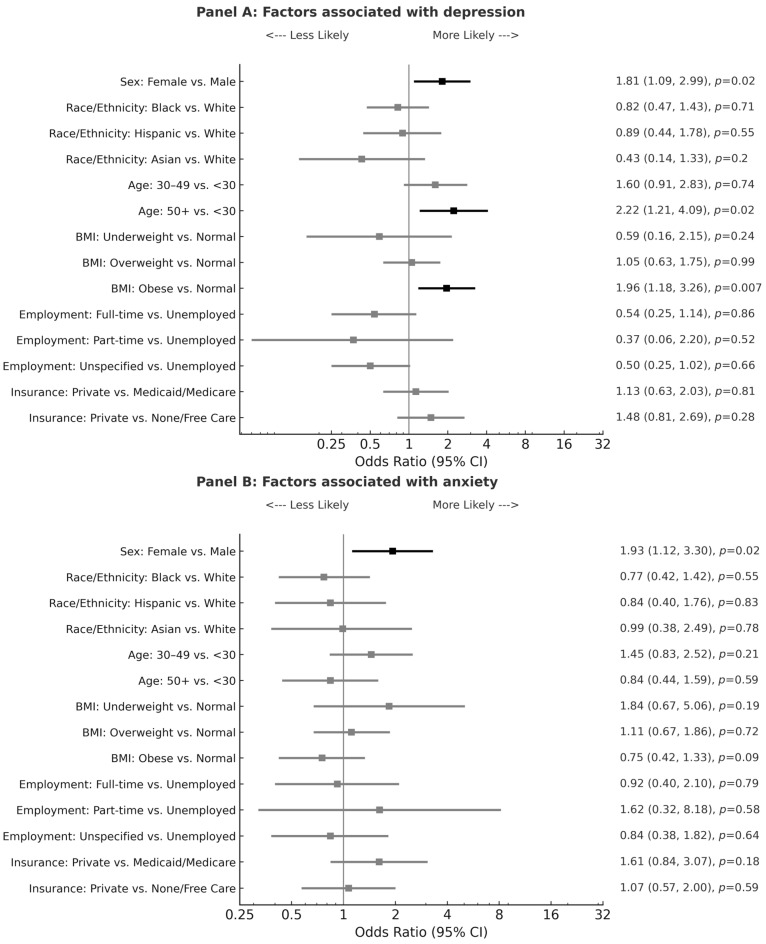
Forest plots for factors associated with depression and anxiety by multiple logistic regression analysis. Forest plots presenting odds ratios (ORs) and 95% confidence intervals (CIs) for demographic, socioeconomic, and clinical factors associated with depression (**Panel A, top**) and anxiety (**Panel B, bottom**). An OR > 1 indicates higher odds relative to the reference group. BMI is body mass index; Underweight: BMI < 18.5; Normal: BMI between 18.5 and 24.9; Overweight: BMI between 25 and 29.9; Obese: BMI ≥ 30. Unspecified employment also includes students. OR ratio bars are depicted in black for significant values.

In addition to having previously identified that the most prevalent concomitant conditions in this IBS cohort include GERD, depression, anxiety, COPD/asthma, and obesity [18], we further investigated the highly comorbid psychiatric conditions of depression and anxiety. Table 2a demonstrates both the significant female prevalence of depression (*p* = 0.008) and anxiety (*p* = 0.03) as individual conditions and as a combined comorbidity (*p* = 0.006) in this IBS population. While rates of depression were similar across major racial/ethnic groups (Table 2b; *p* = 0.77), anxiety was found to be significantly more prevalent in White females compared with non-White female patients with IBS (Table 2b; *p* = 0.02). Prompted by this significant finding, we proceeded to further examine both depression and anxiety by logistic regression analysis. As shown in Figure 1, in addition to female sex, older age and higher BMI were also identified as risk factors for comorbid depression, but not comorbid anxiety. Patients aged fifty years or older with IBS had more than twice the odds for depression compared with patients below the age of thirty (Figure 1; *p* = 0.02). Similarly, IBS patients who were obese had a twofold risk for comorbid depression compared with patients with normal weight (Figure 1; *p* = 0.007). There was no significant association of depression or anxiety with race, employment status, or insurance in this IBS population.

The prevalence rates of eating disorders such as anorexia nervosa (1.3%) and bulimia nervosa (1%) in this IBS cohort exceeded that of the general U.S. population, which is reported by the National Institute of Mental Health (NIMH) to be approximately 0.6% and 0.3%, respectively [35]. In this study, comorbid eating disorders were identified exclusively in White female IBS patients (*n* = 15), except in one Black female patient However, the small number of patients with eating disorders (*n* = 16) in this IBS cohort limited hypothesis testing and only descriptive data were reported (Supplementary Table S4).

### 3.3. IBS Subtypes

**Figure 2:**
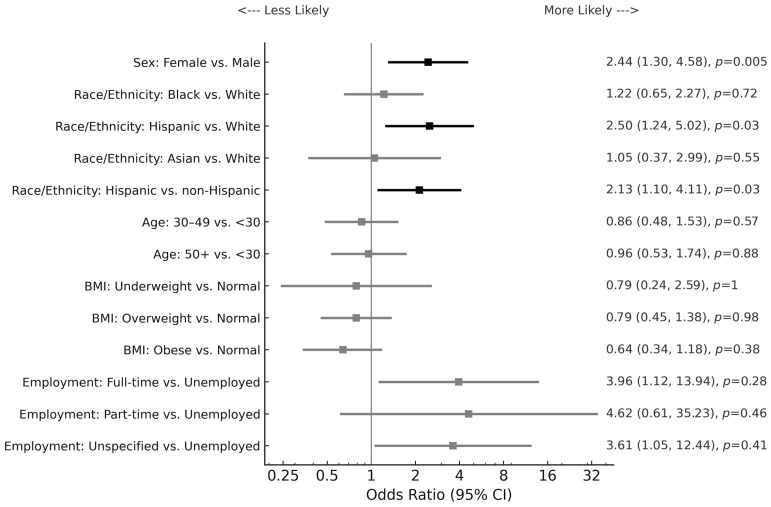
Forest plot for factors associated with IBS with constipation by multiple logistic regression analysis. A forest plot showing ORs and 95% CIs for demographic, socioeconomic, and clinical predictors of IBS with constipation compared with non-constipation IBS subtypes. An OR > 1 reflects higher odds of constipation-predominant IBS relative to the reference group. Non-Hispanic race/ethnicity includes White, Black, and Asian. BMI is body mass index; Underweight: BMI < 18.5; Normal: BMI between 18.5 and 24.9; Overweight: BMI between 25 and 29.9; Obese: BMI ≥ 30. Unspecified employment also includes students. OR ratio bars are depicted in black for significant values.

There were significant sex-related and racial differences identified in the IBS subtype distribution for this IBS sample. For this analysis, the IBS-C and IBS-D patient groups were compared against the corresponding non-constipation (IBS-D, IBS-M, and IBS-U) and non-diarrhea (IBS-C, IBS-M, and IBS-U) groups, respectively. The results are shown in Table 3 and demonstrate that the female patients were more likely to exhibit IBS-C (*p* = 0.03), while the male patients were more likely to have the IBS-D subtype (*p* = 0.03). Logistic regression analysis showed that Hispanic patients were at 2.5 times the risk for IBS-C compared with White patients and at a twofold risk for IBS-C compared with non-Hispanic patients (Figure 2; *p* = 0.03). There was also a significant risk of IBS-C for female patients compared with male patients (Odds ratio = 2.4; *p* = 0.005).

### 3.4. IBS Gastrointestinal and Extraintestinal Comorbidities

In a prior study, we showed that the most common comorbidities within this IBS cohort included GERD (30%), depression (27%), anxiety (23%), COPD/asthma, (16%), and obesity (10%) [18]. This study further investigated the prevalence rates, sex-based differences, and racial/ethnic differences of the selected IBS comorbidities. No sex-related and racial/ethnic differences were identified for GERD, peptic ulcer disease (PUD), non-ulcer dyspepsia (NUD), diverticulosis, constipation, diabetes mellitus, hypercholesterolemia, or COPD/asthma (Supplementary Tables S1–S3). GERD was the most common comorbid condition, occurring in approximately one in three patients within this IBS cohort, which exceeded the estimated prevalence rate of 20% in the general U.S. population [36]. As shown in Table S4, the prevalence rates of Crohn’s disease (0.6%) and ulcerative colitis (0.7%) in this IBS cohort were found to be similar to the overall prevalence rate of inflammatory bowel disease (IBD) in the general national population, which is estimated by the Centers for Disease Control and Prevention (CDC) to range between 0.7% and 0.9% [37]. However, the small sample size of patients with IBD in this study limited hypothesis testing and only descriptive data were reported (Supplementary Table S4).

## 4. Discussion

Irritable bowel syndrome is a highly prevalent chronic, relapsing disorder associated with a significant reduction in health-related quality of life, decreased work productivity, and increased healthcare utilization [2,15]. Prior studies have established that DGBI is typically female predominant, and for IBS particularly, there is a reported female-to-male ratio of 2–2.5:1 based on those who seek medical care [38,39]. Despite this well-recognized sex discrepancy in the prevalence rates of IBS, there is surprisingly very limited knowledge available regarding differences in the comorbid conditions between male and female patients with IBS, specifically as those relate to different racial/ethnic groups.

To our knowledge, this is the first study to investigate sex-related differences in the demographic characteristics and prevalence rates of common comorbidities in patients with IBS at a multiracial inner city safety-net hospital. This is also the first study to perform a direct comparison of sex-based differences in IBS patients from major racial/ethnic groups within one cohort, which represents a unique strength of our study results. Our data highlighted that the psychiatric comorbidities anxiety and depression, which have been previously shown to clinically impact IBS symptom severity [24,25,26], are more prevalent in IBS patients compared with the general population and occur more frequently in female IBS patients than in their male counterparts. This study also demonstrated that anxiety is more prevalent in White female patients compared with non-White female patients with IBS. Additional risk factors identified for comorbid depression in IBS patients included higher BMI and older age. These findings are clinically important because it is essential for clinicians to recognize at-risk subgroups in order to improve patient outcomes by initiating the prompt treatment of both IBS and the identified concurrent conditions. For instance, clinical screening strategies can be implemented including the preclinic administration of psychiatric questionnaires such as the Patient Health Questionnaire-9 (PHQ-9) for depression and the Generalized Anxiety Disorder 7-item (GAD-7) for anxiety. Subsequently, appropriate referrals to psychiatric specialists can be offered and submitted for patients with positive assessments, thereby facilitating the timely diagnosis and management of mood disorders. Previous studies have indicated that psychological factors have a cumulative effect on gastrointestinal symptoms as well as quality of life in patients with IBS [24,25,26].

Within this cohort of 740 IBS outpatients at a diverse tertiary care center, the male and female patients were similar in most demographic characteristics, but the female patients had a lower BMI (*p* = 0.03), more commonly presented in a younger age group (*p* = 0.03), and were more likely to be current smokers (*p* = 0.03) compared with the male patients. These findings are consistent with prior studies, including a cross-sectional study by Almario et al. that found that female individuals and every day smokers had higher odds for IBS-C, IBS-D, and IBS-M [8]. In addition to demographics, our study also examined the distribution of sex-based differences for concomitant extraintestinal and gastrointestinal conditions in patients with IBS.

The association between IBS and psychiatric conditions has garnered increasing interest from researchers, with some studies indicating that up to 60% of IBS patients suffer from psychiatric disorders, the most common being anxiety, depression, panic, posttraumatic stress, and somatization disorders [40]. In general, depressive disorders are recognized as leading causes of disease burden worldwide, with devastating economic and social consequences [41]. According to the World Health Organization (WHO), approximately 5% of the global population experience depression, with an estimated depression rate of 4% among males and 6% among females [42]. In a breakdown of U.S. state estimates for a lifetime diagnosis of depression, the CDC reported a prevalence rate of 17.9% for the jurisdiction of Massachusetts compared with a rate of 26% in our IBS study cohort at the Boston Medical Center (BMC) [43], the largest safety-net hospital in New England, serving a diverse patient population from Boston and nearby communities. The WHO also reported that approximately 4% of the global population have anxiety disorders [44]. Epidemiological studies have estimated that the worldwide prevalence rate of anxiety is 3% in males and 5% in females [45]. The overall female predominance of depression and anxiety may be attributed to numerous factors including the sex-based variability of genetic and epigenetic predisposition, hormonal milieu, and neuroplasticity [41,46,47]. Our study highlights that depression and anxiety are highly comorbid in all IBS patients, with rates that substantially exceed those in the general population. The reason behind this strong correlation remains to be fully understood and warrants the elucidation of how depression might increase the symptom severity in IBS.

Dysregulation of gut–brain pathways has been implicated in the pathogenesis of IBS [48,49]. The gut–brain axis is a theoretical model outlining the bi-directional communication between the enteric and central nervous systems [50]. Prior studies have suggested that psychosocial and environmental stressors can impact IBS via neural, immunological, and endocrine pathways [51,52]. However, the intricacies of these interactions remain elusive. There are contending theories depicted by two biopsychological models of IBS: the top-down model and the bottom-up model [19,53]. The top-down model proposes that psychosocial stressors influence physiological mechanisms including sensory processing, motor functions, and stress reactivity of the gut via vagal and sympathetic afferents [19,53]. Conversely, the bottom-up model suggests that IBS symptoms result from interoceptive stresses of the gut, which can subsequently contribute to anxiety and depression [19,53]. While it remains unclear whether symptoms of IBS elicit anxiety and depression or if these affective disorders induce dysfunction of the brain–gut pathways, our data support that the presence of anxio-depressive symptoms, particularly in female individuals, is a risk factor for IBS. Therefore, recognizing anxiety and depression in patients with IBS and treating both symptomologies of IBS and psychiatric disorders concurrently can significantly improve patient quality of life.

For patients with IBS, various factors have been shown to impact health-related quality of life. In a cross-sectional study, Creed et. al. evaluated IBS patients using the Symptom Checklist 90 Revised (SCL-90R) questionnaire and the Hamilton rating scale of depression, which revealed that psychological distress was an independent and significant predictor of health-related quality of life in addition to the severity of gastrointestinal symptoms [54]. Similarly, Van Tilburg et al. conducted a cross-sectional study using validated questionnaires (IBS Symptom Severity Scale (IBS-SSS), Brief Symptom Inventory-18 (BSI-18), Coping Strategies Scale) and determined that anxiety indirectly impacted IBS severity through various maladaptive coping mechanisms such as catastrophizing and somatization [55]. Given that our current study demonstrated the substantial prevalence of anxio-depressive symptoms in female patients with IBS, exceeding that in the general population, these results collectively suggest that female IBS patients with anxiety are at risk of increased IBS severity and lower health-related quality of life. However, it should be noted that the retrospective nature of this current study prevents the determination of cause and effect, and this study also did not assess the quality of life in these patients.

This study highlights that in addition to female sex, other major risk factors for comorbid depression in patients with IBS include older age and higher BMI (Figure 1). The association between older age and depression may simply be explained by the higher disease burden given the increased number of years that patients are afflicted with IBS symptoms. Furthermore, through a metabolomic and metagenomic profiling of stool and serum samples of IBS patients, Han et al. proposed a correlation between tryptophan (TRP)/serotonin metabolism and IBS depression comorbidity [56]. To date, there are three major studied pathways of gut TRP metabolism that produce serotonin, kynurenine (KYN), and indole derivatives, respectively. Within the gut microbiome, certain bacteria strains have been shown to directly regulate TRP utilization and impact serotonin levels in both the periphery and the brain [56]. In instances when TRP metabolism is diverted to KYN derivatives, there is a subsequent deficiency of TRP in the brain that results in decreased neurotransmitter serotonin production, which may ultimately contribute to depression [56]. Interestingly, in a study by Chaves Filho et al., KYN was also implicated in a shared pathway between depression and obesity [57]. This aligns with our findings that IBS patients who are obese are at increased risk for depression (Figure 1). Collectively, these results suggest the possibility of a shared role of TRP metabolism underpinning the comorbidity between IBS, depression, and obesity. However, the retrospective nature of this current study precludes causal conclusions.

To our knowledge, this study is the first to investigate sex-based differences in the depression and anxiety rates across major racial/ethnic groups in patients with IBS. The rates of depression were found to be uniformly higher in females compared with male patients at 29% and 19%, respectively, without significant differences across racial/ethnic groups. While rates of anxiety were also collectively higher in females compared with males with IBS at 24% and 17%, respectively, anxiety was significantly more prevalent in White female patients compared with non-White female patients (*p* = 0.02).

This racial discrepancy of anxiety rates in female patients with IBS is likely multifactorial and may be in part due to racial disparities in healthcare utilization. A cross-sectional study by Burnett-Zeigler et al. reported lower rates of participation in psychiatric services among Black and Hispanic patients compared with White patients over a 12-month period [58]. Additionally, another study demonstrated that minority patients (Black, Hispanic, and Asian) were less frequently referred for specialty visits despite IBS symptoms compared with White patients [59]. Some studies have also suggested that minority patients harbor higher levels of mistrust in the healthcare system and are less likely to seek medical care, resulting in potential delays in the diagnosis and treatment of various conditions [60]. It is essential to recognize these racial discrepancies in the prevalence rates, diagnoses, and treatment of psychiatric and medical conditions in order to improve the health outcomes for all IBS patients.

In addition to depression and anxiety, this study also assessed for other comorbid psychiatric conditions among IBS patients. While it has been suggested that disordered eating habits can often co-occur in patients with IBS, particularly those with increased stress and anxiety [61,62], the relationship between eating disorders and DGBI remains largely unexplored. In this current study, a small subset of IBS patients was identified to have eating disorders, namely anorexia nervosa and anorexia bulimia. Mirroring trends observed in the general population [35], both eating disorders in this study appeared to be more prevalent in female patients compared with male patients with IBS—in fact, eating disorders were exclusively confined to female patients in this study cohort (Table S4). However, despite observable sex-based differences, the sample sizes of the study patients with anorexia nervosa and anorexia bulimia were too small for hypothesis testing. Similar to the notable racial differences in the prevalence rates of anxiety within this IBS cohort, eating disorders appeared to be more prevalent in White female compared with non-White female patients (Table S4), but the statistical significance of this finding could not be determined due to the small number of affected patients. Interestingly, comorbid bipolar disorder, schizophrenia, and personality disorders were found very rarely in IBS patients, and in contrast to eating disorders, with apparent similar frequencies between female and male patients. However, this study was limited and did not permit for meaningful hypothesis testing of the sex-based or ethnic/racial differences.

We previously identified several gastrointestinal and extraintestinal conditions that commonly co-occurred with IBS, with GERD identified as the most prevalent comorbidity [18]. GERD occurred in 30% of all study participants, which is aligned with the previously reported range of 10–74% for a GERD-IBS overlap based on endoscopic diagnosis [63,64]. In this current study, we found no substantial difference in the prevalence rates of GERD, diabetes mellitus, PUD, NUD, diverticulosis, or constipation between the male and female patients (Supplementary Table S3).

There were, however, significant sex-related and racial/ethnic differences in the IBS subtype distribution. Female patients were found to have more IBS-C compared with male patients at 23% and 16%, respectively (Table 3). This finding is concordant with a secondary analysis of a clinical trial that suggested that IBS-D is more common in males, whereas IBS-C is more common in female individuals [31]. Similarly, another study reported that IBS-C, IBS-M, and IBS-U were more common in female patients [65]. Such sex-based differences in the clinical presentation of IBS are likely multifactorial and a result of several mechanisms underlying visceral sensitivity, gastrointestinal processing, and hormonal differences with gut function [66]. Previous data from animal studies have implicated the role of hormones in both visceral sensitivity and GI motility; estrogen and progesterone were found to slow gastric emptying and modulate sensitivity to pain and temperature, while testosterone exhibited no significant effect on GI motility [67,68]. Of note, prior studies have indicated that the healthcare utilization rates were influenced by sex and IBS-subtype, with increased healthcare costs associated with female sex and IBS-C subtype [11].

Further analysis of our data with multivariate logistic regression analysis revealed that Hispanic patients had more than twice the risk for IBS-C compared with non-Hispanic patients (*p* = 0.03). Studies examining the association between IBS and race/ethnicity are very scarce and available data are inconsistent. Prior results from Cheng et al. found that Hispanic patients had a higher frequency of IBS-C when compared with non-Hispanic IBS patients at 32% and 20%, respectively [32]. Conversely, Zuckerman et al. found no difference in IBS-type symptoms between Hispanic and non-Hispanic White patients [69].

While this study offers several novel insights into the sex-related and racial/ethnical differences in IBS and enrolled a relatively large number of participants, we need to acknowledge certain limitations of this study. It was a retrospective design of the study, and patient enrollment was based on the ICD-9 coding for IBS. Even though chart reviews were performed with high diligence and substantial inter-rater agreement, diagnoses could not be confirmed with the validated IBS questionnaires due to the retrospective design of this study, and patients could have been potentially misclassified in the electronic medical record system, which may have introduced potential selection bias. Similarly, the IBS subtype classification was based upon the documented clinical presentation and chart reviews rather than validated questionnaires. Future research efforts should include replicating this study using the most recently updated classification and coding systems such as the Rome IV criteria and ICD-10 coding. Prior studies have found lower IBS prevalence rates when applying the Rome IV compared with the Rome III criteria, suggesting the more restrictive diagnostic criteria of the Rome IV compared with the previous reference standard for IBS [70,71]. Additionally, the IBS subtype classification may be improved by using the ICD-10 instead of ICD-9 coding because ICD-10 now encompasses more codes including various IBS subtypes. While data collection occurred between 2005 and 2007 during the Rome III era of IBS diagnosis, the quality and significance of the reported data provide critical and previously unknown epidemiological information across different racial groups from a single institution with a large diverse patient population. While the data were derived from the Rome III era, it is by no means outdated, rather, they significantly contribute to fill substantial contemporary knowledge gaps. Furthermore, this study presents unique data during a time when the insurance coverage of patients with IBS largely included free care, which has since been changed in the state of Massachusetts to a mandatory government funded MassHealth [72]. Therefore, it appeared particularly compelling to collect and analyze data originating in a timeframe prior to this change. Given the time since initial data collection, future research endeavors should include replicating this study and comparing the results with that of a new cohort under current insurance mandates and updated IBS classification systems under the ICD-10 coding. Additionally, the various comorbidities investigated in this study were not comprehensive and did not include other commonly encountered IBS comorbid conditions such as fibromyalgia and chronic fatigue syndrome, which should be remedied in future studies. Future studies should also systematically assess the impact of socioeconomic status, cultural factors, and healthcare access on sex-related differences in IBS.

## 5. Conclusions

In conclusion, we presented a study examining the sex-based differences in a racially/ethnically diverse outpatient IBS population with several novel, clinically important findings. We determined the presence of significant sex-based and racial differences related to BMI, age, IBS subtype distribution, and comorbid anxiety and depression. Anxiety and depression were highly comorbid in all IBS patients, with prevalence rates substantially surpassing those of the general population. We identified that significant risk factors for comorbid depression include older age, higher BMI, and female sex, but not race. Given the high disease burden of DGBI and associated detrimental impact on patient quality of life, the findings of this study provide useful information for clinicians to recognize risk factors for IBS, treat common comorbidities, and ultimately improve health outcomes by targeting risk factors in IBS management.

## Figures and Tables

**Table 1:** Demographic characteristics of male and female patients with IBS.

Characteristics	All IBS Patients (*n* = 740)	Male Patients (*n* = 185)	Female Patients (*n* = 555)	*p* Value
Median age, years [range]	40 [19–89]	42 [19–89]	40 [19–88]	0.11
Median age at IBS dx, years [range] ^a^	33 [10–89]	34 [11–89]	32 [10–83]	0.75
Median BMI [range] ^b^	25.4 [14.3–64.2]	26.6 [14.3–44.4]	25.0 [15.7–64.2]	0.03
BMI range, *n* (%):				0.01
Underweight (<18.5)	18 (3%)	3 (2%)	15 (4%)	
Normal (18.5–24.9)	227 (43%)	43 (33%)	184 (46%)	
Overweight (25–29.9)	153 (29%)	53 (41%)	100 (25%)	
Obese (≥30)	129 (24%)	30 (23%)	99 (25%)	
Current age, *n* (%):				0.03
<30	196 (27%)	35 (19%)	161 (29%)	
30–49	298 (40%)	83 (45%)	215 (39%)	
50+	246 (33%)	67 (36%)	179 (32%)	
Race/ethnicity, *n* (%)				0.07
White	492 (70%)	112 (64%)	380 (72%)	
Black	99 (14%)	24 (14%)	75 (14%)	
Hispanic	65 (9%)	22 (13%)	43 (8%)	
Asian	44 (6%)	16 (9%)	28 (5%)	
Not reported (*n* = 40) ^c^	—	—	—	
Marital status, *n* (%):				0.09
Married	238 (33%)	73 (40%)	165 (31%)	
Single	401 (56%)	95 (52%)	306 (57%)	
Divorced	77 (11%)	14 (8%)	63 (12%)	
Widowed	6 (1%)	1 (1%)	5 (1%)	
Not reported (*n* = 18) ^d^	—	—	—	
With children, *n* (%):				0.98
Yes	191 (48%)	50 (48%)	141 (48%)	
No	207 (52%)	54 (52%)	153 (52%)	
Not reported (*n* = 342) ^e^	—	—	—	
Smoking Hx, *n* (%):				0.03
Current	106 (17%)	23 (14%)	83 (19%)	
Former	100 (16%)	37 (23%)	63 (14%)	
Never	403 (66%)	104 (63%)	299 (67%)	
Not reported (*n* = 131) ^f^	—	—	—	
Insurance type, *n* (%):				0.56
Private insurance	507 (69%)	121 (65%)	386 (70%)	
Medicaid/Medicare	123 (17%)	33 (18%)	90 (16%)	
None/Free Care	110 (15%)	31 (17%)	79 (14%)	
Highest level of education, *n* (%):				0.005
Pre high school	3 (1%)	2 (3%)	1 (1%)	
High school	15 (7%)	8 (13%)	7 (5%)	
College	84 (41%)	15 (25%)	69 (48%)	
Graduate/Professional	103 (50%)	36 (59%)	67 (47%)	
Not reported (*n* = 535) ^g^	—	—	—	
Employment, *n* (%):				0.04
Unemployed	55 (7%)	15 (8%)	40 (7%)	
Full time	214 (29%)	68 (37%)	146 (26%)	
Part time	19 (3%)	3 (2%)	16 (3%)	
Unspecified *	452 (61%)	99 (54%)	353 (64%)	

Not reported data for: ^a^ Age at irritable bowel syndrome (IBS) diagnosis: *n* = 72 for male and *n* = 231 for female; ^b^ Body mass index (BMI): *n* = 56 for male and *n* = 157 for female; ^c^ Race/ethnicity: *n* = 10 for male and *n* = 29 for female; 1 Native American male was not included. Analyses based on race/ethnicity were conducted in a cohort of 700 patients. Not reported data for: ^d^ Marital status: *n* = 2 for male and *n* = 16 for female; ^e^ Children status: *n* = 81 for male and *n* = 261 for female; ^f^ Smoking history: *n* = 21 for male and *n* = 110 for female; ^g^ Highest level of education: *n* = 124 for male and *n* = 411 for female. * Unspecified employment also includes students. BMI is kg body weight/body surface area in m^2^. The sum of percentages in some groups did not add up to exactly 100% as a result of rounding.

**Table 2:** (a) Depression and anxiety in male and female IBS patients; (b) Depression and anxiety in racially/ethnically diverse IBS patients.

(**a**)				
	All IBS (*n* = 740)	Male (*n* = 185)	Female (*n* = 555)	*p* value
Depression	195 (26%)	35 (19%)	160 (29%)	0.008
Anxiety	166 (22%)	31 (17%)	135 (24%)	0.03
Depression and anxiety *	80 (11%)	14 (8%)	66 (12%)	0.006
(**b**)				
		Male	Female	*p* value
Depression ^a^				
White		24 (71%)	104 (68%)	0.77
Non-White		10 (29%)	49 (32%)	
Anxiety ^b^				
White		16 (53%)	97 (75%)	0.02
Non-White		14 (47%)	32 (25%)	
Depression and anxiety ^c^				
White		10 (77%)	45 (70%)	0.75
Non-White		3 (23%)	19 (30%)	

* Concurrent depression and anxiety. ^a^ *n* = 34 for male and *n* = 153 for female; ^b^ *n* = 30 for male and *n* = 129 for female; ^c^ *n* = 13 for male and *n* = 64 for female.

**Table 3:** Comparison of IBS subtypes between male and female patients.

	Male (*n* = 185)	Female (*n* = 555)	*p* Value
IBS with constipation			
Yes	29 (16%)	130 (23%)	0.03
No *	156 (84%)	425 (77%)	
IBS with diarrhea			
Yes	58 (31%)	129 (23%)	0.03
No **	127 (69%)	426 (77%)	

* No IBS with constipation includes IBS with diarrhea, IBS with mixed bowel habits, and unsubtyped IBS. ** No IBS with diarrhea includes IBS with constipation, IBS with mixed bowel habits, and unsubtyped IBS.

## Data Availability

The original contributions presented in the study are included in the article; further inquiries can be directed to the corresponding author/s.

## Supplementary Materials

The following supporting information can be downloaded at: https://www.mdpi.com/article/10.3390/diseases13050161/s1, Table S1: Selected gastrointestinal comorbidities in a racially/ethnically diverse outpatient population with IBS; Table S2: Selected non-gastrointestinal comorbidities in a racially/ethnically diverse outpatient population with IBS; Table S3: Sex-based estimated prevalence rates of selected gastrointestinal and non-gastrointestinal comorbidities in a racially/ethnically diverse outpatient population with IBS; Table S4: Eating disorders and inflammatory bowel disease (IBD) in a racially/ethnically diverse outpatient population with IBS.

## Abbreviations

The following abbreviations are used in this manuscript:

IBS	Irritable bowel syndrome
IBS-C	IBS with constipation
IBS-D	IBS with diarrhea
IBS-M	IBS with mixed bowel habits
IBS-U	Un-subtyped IBS
DGBI	Disorder of gut–brain interaction
FGID	Functional gastrointestinal disorder
HRQoL	Health-related quality of life
GERD	Gastroesophageal reflux disease
PUD	Peptic ulcer disease
NUD	Non-ulcer dyspepsia
ESRD	End-stage renal disease
COPD	Chronic obstructive lung disease
WHO	World Health Organization
CDC	Centers for Disease Control and Prevention
NIMH	National Institute of Mental Health
ICD-9	International Classification of Diseases, 9th Revision
ICD-10	International Classification of Diseases, 10th Revision
BMC	Boston Medical Center
PHQ-9	Patient Health Questionnaire-9
GAD-7	Generalized Anxiety Disorder 7-item
HHS	Department of Health and Human Services
IBS-SSS	IBS Symptom Severity Scale
BSI-18	Brief Symptom Inventory-18
