# Supplementary material for: Effects of Sex and Race on Epidemiology and Comorbidities of Patients with Irritable Bowel Syndrome: A Rome III Era Retrospective Study
Source: Diseases. 2025 May 21;13(5):161. doi: 10.3390/diseases13050161 (PMC12110308; doi:10.3390/diseases13050161)
Supplement: Supplementary file 1 [file diseases-13-00161-s001.zip › diseases-3606634-supplementary.pdf]

Supplemental Tables:

Table S1. Selected Gastrointestinal Comorbidities in a Racially/Ethnically Diverse Outpatient Population with IBS

	**GERD (N=207)**		
Racial/Ethnic group	Male (N=51)	Female (N=156)	P value
			0.36
White	34	97	
Black	5	28	
Hispanic	8	25	
Asian	4	6	
	**PUD* (N=18)**		
	Male (N=5)	Female (N=13)	
			0.45
White	3	9	
Black	1	4	
Hispanic	1	0	
Asian	0	0	
	**NUD** (N=48)**		
Racial/Ethnic group	Male (N=11)	Female (N=37)	
			0.07
White	3	20	
Black	3	10	
Hispanic	1	5	
Asian	4	2	
	**Diverticulosis (N=35)**		
Racial/Ethnic group	Male (N=9)	Female (N=26)	
			0.54
White	8	17	
Black	0	5	
Hispanic	1	3	
Asian	0	1	
	**Constipation (N=58)**		
Racial/Ethnic group	Male (N=15)	Female (N=43)	
			0.55
White	7	27	
Black	3	8	
Hispanic	4	6	
Asian	1	2	

Comparison of gastrointestinal comorbidities by sex and race. The total IBS sample (N=740) is comprised of 185 male and 555 female patients (see Table 1). Based on not reported data for race (N=39; 29 females and 10 males) and one Native American male patient that was not included, this sample is comprised of 700 patients (see Table 1).
*PUD is Peptic Ulcer Disease; **NUD is Non-Ulcer Dyspepsia; IBS is Irritable Bowel Syndrome.

Table S2. Selected Non-Gastrointestinal Comorbidities in a Racially/Ethnically Diverse Outpatient Population with IBS

	**Diabetes Mellitus (N=38)**		
	Male (N=9)	Female (N=29)	P value
			1.0
White	5	13	
Black	3	11	
Hispanic	1	4	
Asian	0	1	
	**Hypercholesterolemia (N=123)**		
Racial/Ethnic group	Male (N=32)	Female (N=91)	
			0.21
White	16	62	
Black	9	18	
Hispanic	5	9	
Asian	2	2	
	**COPD/ Asthma (N=112)**		
Racial/Ethnic group	Male (N=28)	Female (N=84)	
			0.73
White	19	56	
Black	3	15	
Hispanic	4	9	
Asian	2	4	

Comparison of non-gastrointestinal comorbidities by sex and race in IBS. The total IBS sample (N=740) is comprised of 185 male and 555 female patients (see Table 1). Based on not reported data for race (N=39; 29 females and 10 males) and one Native American male patient that was not included, this sample is comprised of 700 patients (see Table 1). IBS is Irritable Bowel Syndrome.

Table S3. Sex-Based Estimated Prevalence Rates of Selected Gastrointestinal and Non-Gastrointestinal Comorbidities in a Racially/Ethnically Diverse Outpatient Population with IBS

	Gastrointestinal Comorbidities		
	Male (N=185)	Female (N=555)	P value
GERD			?
Yes	55 (30%)	169 (30%)	
No	130 (70%)	386 (70%)	
PUD			?
Yes	6 (3%)	14 (3%)	
No	179 (97%)	541 (97%)	
NUD			?
Yes	12 (6%)	40 (7%)	
No	173 (94%)	515 (93%)	
Diverticulosis			?
Yes	9 (5%)	27 (5%)	
No	176 (95%)	528 (95%)	
Constipation			?
Yes	15 (8%)	48 (9%)	
No	170 (92%)	507 (91%)	
	Non-Gastrointestinal Comorbidities		
	Male (N=185)	Female (N=555)	P value
Diabetes Mellitus			?
Yes	10 (5%)	30 (5%)	
No	175 (95%)	525 (95%)	
Hypercholesterolemia			?
Yes	37 (20%)	94 (17%)	
No	148 (80%)	461 (83%)	
COPD/ Asthma			?
Yes	28 (15%)	87 (16%)	
No	157 (85%)	468 (84%)	

The total IBS sample (N=740) is comprised of 185 male and 555 female patients (see Table 1).

Table S4. Eating Disorders and Inflammatory Bowel Disease (IBD) in a Racially/Ethnically Diverse Outpatient Population with IBS

	**Anorexia Nervosa (N=9)**	
Racial/Ethnic group	Male (N=0)	Female (N=9)
White	0	9
Black	0	0
Hispanic	0	0
Asian	0	0
	**Bulimia Nervosa (N=7)**	
Racial/Ethnic group	Male (N=0)	Female (N=7)
White	0	6
Black	0	1
Hispanic	0	0
Asian	0	0
	**Eating Disorders* (N=15)**	
Racial/Ethnic group	Male (N=0)	Female (N=15)
White	0	15
Black	0	1
Hispanic	0	0
Asian	0	0
	**Bipolar Disorder (N=14)**	
Racial/Ethnic group	Male (N=3)	Female (N=11)
White	3	9
Black	0	2
Hispanic	0	0
Asian	0	0
	**Schizophrenia (N=5)**	
Racial/Ethnic group	Male (N=1)	Female (N=4)
White	1	1
Black	0	2
Hispanic	0	1
Asian	0	0
	**Personality Disorders (N=8)**	
Racial/Ethnic group	Male (N=1)	Female (N=7)
White	1	6
Black	0	1
Hispanic	0	0
Asian	0	0
	**Crohn's Disease (N=4)**	
Racial/Ethnic group	Male (N=1)	Female (N=3)
White	1	2
Black	0	1
Hispanic	0	0
Asian	0	0
	**Ulcerative Colitis (N=5)**	
Racial/Ethnic group	Male (N=1)	Female (N=4)
White	1	4
Black	0	0
Hispanic	0	0
Asian	0	0

Comparison of eating disorders and IBD by sex and race in IBS. The total IBS sample (N=740) is comprised of 185 male and 555 female patients (see Table 1). Based on not reported data for race (N=39; 29 females and 10 males) and one Native American male patient that was not included, this sample is comprised of 700 patients (see Table 1). Due to small sample size, hypothesis testing was not performed. IBD is Inflammatory Bowel Disease; IBS is Irritable Bowel Syndrome.
